# Effects of Continuous Carbohydrate Intake with Gummies during the Golf Round on Interstitial Glucose, Golf Performance, and Cognitive Performance of Competitive Golfers: A Randomized Repeated-Measures Crossover Design

**DOI:** 10.3390/nu15143245

**Published:** 2023-07-21

**Authors:** Yosuke Nagashima, Kiyohiro Ehara, Yoshitomo Ehara, Ayana Mitsume, Kie Kubo, Shigeru Mineo

**Affiliations:** 1 Department of Health Science, Musashigaoka Junior College, 111-1 Minamiyoshimi, Saitama 355-0154, Japan; mitsume-a@musashigaoka.ac.jp; 2 Department of Education, Tamagawa University, Tokyo 194-8610, Japan; ehrky0ot@stu.tamagawa.ac.jp; 3 Research Institute of Wellness, Rikkyo University, Tokyo 171-8501, Japan; y-ehara@rikkyo.ac.jp; 4 Freelance Dietitian, Saitama 369-0311, Japan; kkubo.2929@gmail.com; 5 Nutraceuticals Science Laboratory, Advanced Research Institutes, Bourbon Corporation, Niigata 945-8611, Japan; mineo-shi@bourbon.co.jp

**Keywords:** sport nutrition, sport performance, golf, carbohydrates, interstitial glucose, cognitive performance

## Abstract

This study examined the effects of continuous carbohydrate intake during a golf round on interstitial glucose, golf performance, and the cognitive performance of competitive golfers. Eleven competitive golfers participated and played 18 holes of golf in this study. Participants were randomly assigned to the group indicated to consume the test food (CHO intake) or the group required to not consume it (NOT intake). Here, gummies were used as the test food, and the amount of carbohydrates was 30 g per h. Blood glucose levels were evaluated using interstitial glucose. Golf performance was measured in five tests, including scores, 2.5 m putting test, club head speed, driving distance, and accuracy. Cognitive performance was measured in three tests, including self-perceived levels of fatigue (PLF), self-perceived levels of concentration (PLC), and self-perceived levels of relaxation (PLR). Interstitial glucose (*p* < 0.001) and PLF (*p* < 0.001) were significantly reduced in the CHO intake compared with that in the NOT intake from the sixth hole. PLC was significantly higher in the CHO intake than in the NOT intake on all 18 holes (*p* = 0.032). These findings suggest that continuous carbohydrate intake may be effective in reducing fatigue and maintaining the performance of competitive golfers.

## 1. Introduction

Golf is reported to be a moderate-intensity sport with a physical activity level of 4.8 METs, with an extended duration of 18 holes (a round) lasting anywhere from 4 to 5 h while covering distances over 10 km [1,2,3,4,5,6]. When played competitively, golf involves high cognitive loads, critical shot-making decisions, hand–eye coordination, high-level motor and biomechanical skills, and an extended duration of play that exceeds most other sports [7,8]. This combination of critical shot-making decisions, multiple maximum effort swings, putting, and long distances of walking during a round can result in physical and mental fatigue [9], i.e., golf-specific fatigue [4], and can negatively affect golf performance.

The American College of Sports Medicine (ACSM) recommends endurance athletes to engage in exercise longer than 1 h and ingest carbohydrates at a rate of 30–60 g per hour to maintain carbohydrate oxidation and delay fatigue [10]. However, previous studies have not examined the effects of ingesting 30–60 g of carbohydrates on golfers. Stevenson et al. (2009) found that the consumption of carbohydrates consumed prior to, and twice during the round on holes 6 and 12, improved both motor performance, measured through 2 m and 5 m putts, and cognitive performance: self-rated scores of alertness and fatigue [4].

Research indicates that hypoglycemia during golf can lead to concentration loss, irritability, impaired judgment [7], cognitive performance acme [11], and high ratings of self-perceived fatigue [12]. Thus, a specific nutrition plan is necessary to maintain euglycemia over 18 holes [4,5]. To develop a nutrition plan for controlling fatigue, symptoms of hypoglycemia and fatigue must be observed.

Until now, collecting a small amount of capillary blood from the fingertip by puncture is a simple method of measuring blood glucose during exercise. Therefore, this measurement method is not possible in ongoing golf play. To solve this problem, continuous glucose monitoring (CGM) for blood glucose measurement was employed in this study. These CGM systems can be easily worn on the body and can automatically and constantly measure the glucose concentration in the interstitial fluid. The glucose concentration in the interstitial fluid of the subcutaneous tissue closely correlates with the glucose concentration in the blood [13,14,15]. The CGM system is minimally invasive [16] and has been used to measure blood glucose in other sports [17,18].

This study aimed to examine the effects of continuous carbohydrate intake during a golf round on interstitial glucose, golf performance, and the cognitive performance of healthy male competitive golfers. The hypothesis is that continuous carbohydrate intake will attenuate the decline in interstitial glucose level and cognitive performance and improve golf performance.

## 2. Materials and Methods

### 2.1. Study Design

**Figure 1:**
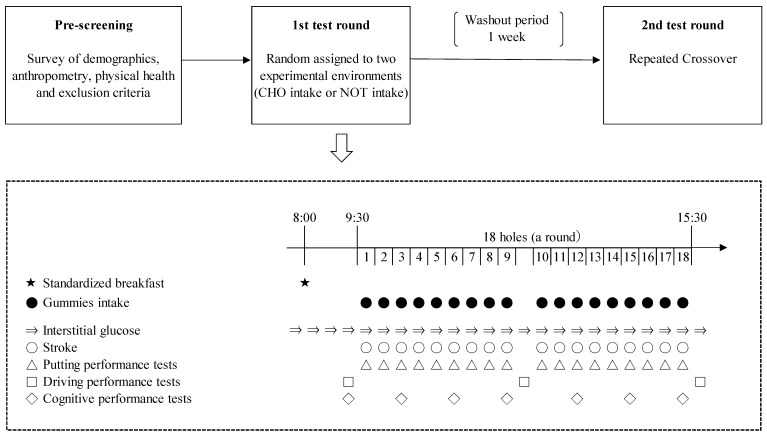
Study Design.

The study design is shown in Figure 1. In a randomized repeated-measures crossover design, each participant was assigned to ingest carbohydrate-supplemented gummies continuously (CHO intake) or not to ingest it (NOT intake) during a golf play in two separate rounds. This study was conducted with maximum real-life application.

In this study, participants were required to show up three times. First, during prescreening, initial data collected included age, participation status in the competition, anthropometry, and physical health, and inclusion and exclusion (intake of healthy foods and smoking) criteria were evaluated. In addition, participants were fitted with a device that can measure the interstitial blood glucose levels. Second, the participants played the 1st test round of golf. Thereafter, the washout period was 1 week. Since the active ingredient in this study was carbohydrates, a duration of ≥6 days from the first intake was considered sufficient. Third, the participants played the 2nd test round of golf.

This study was conducted following the guidelines stipulated in the Declaration of Helsinki, and all procedures were approved by the Ethics Committee of Musashigaoka Junior College, Japan (No. 21–3). In addition, this study was registered in a public database set up by the University Medical and Information Network (Study ID: UMIN000046694).

### 2.2. Participants

A convenience sample of 12 male competitive golfers was recruited from the Kanto Students Golf Association First Division. Based on our initial sample size calculation, we speculated that a sample of at least 12 participants was necessary to achieve a power of 0.80 with an alpha value set at 0.05. The participants were all right-handed. The participant criteria were as follows: consume healthy foods, were nonsmokers, competed in national or regional competitions, and were injury free. Moreover, the participants were informed about the experimental procedures, and they read and signed the informed consent form. If a participant was a minor, written informed consent and assent was obtained from participants (assent) and their parents/guardians (consent). Twelve participants agreed to take part in this study (response rate, 100%). Of these 12 participants, 1 was excluded from the analyses because of missing data (interstitial glucose could not be measured). Ultimately, 11 participants (84.6%) were included in the analysis (age 20.1 ± 1.0 years, body height 173.6 ± 5.9 cm, bodyweight 74.8 ± 13.7 kg, and body fat percentage 21.6 ± 6.4%).

### 2.3. Procedures

This study was conducted at the Ishizaka Golf Club at Hatoyama, Hiki-gun, Saitama, Japan. The study was performed between February and March 2022. Participants were required to abstain from consuming alcohol during the evening before each round and to come to the venue without breakfast on each test day. The participants were required to report to the clubhouse 30 min before their scheduled start time. Thereafter, the participants consumed a standardized breakfast served at Ishizaka Golf Club in the starting order. During both trials, a standardized breakfast (635 kcal: 12% protein, 25.1% fat, and 48.3% carbohydrates), which consisted of rice, miso soup, salted salmon, natto, and boiled egg, was consumed between 8:00 a.m. and 8:30 a.m. on the experiment day. The energy and nutritional intake of breakfast were calculated by a dietitian using the weighing method. Nutritional intake was estimated using Eiyou-plus version 1 (Kenpakusha Co., Ltd., Tokyo, Japan) with the Standard Tables of Food Composition in Japan, 2020 Edition (8th Revision). The ACSM guidelines recommend 1–4 g bodyweight (BW) (intake per kg of BW) of carbohydrates 1–4 h before exercise [10]. The prescribed breakfast in this study contained approximately the same amount of carbohydrates (1.02 kg BW, which met the ACSM reference values).

In this study, gummies (Fettuccine Gummi, Bourbon Corporation, Niigata, Japan) were used as the test food during the golf round. In Japan, gummies are commonly distributed and readily available food; in addition, it is rich in carbohydrates. Thus, in this study, we considered gummies to be a suitable food for competitive golfers in terms of real-life applications. The recommended carbohydrate intake in endurance competitions is 30–60 g per h [10,19]. In golf competitions, if the time required for 18 holes is 5 h, 150–300 g of carbohydrates is needed. In this study, one gummy contained 2.3 g of carbohydrates (0.1 g of dietary fiber and 2.2 g of sugars). Thus, the participants were required to eat a total of 72 gummies, totaling at least 150 g of carbohydrates. Before their first round, participants were randomly allocated to the CHO intake or the NOT intake. The CHO intake consumed four gummies per hole (total 72 gummies). In the CHO intake, the accompanying researcher offered 12 gummies for every three holes. The CHO intake was requested to chew them well, aiming for 30 bites per mouthful. However, there was no limit to the timing of ingestion. No study participant failed to eat the prescribed number of gummies. In addition, all participants were provided with water (two 500 mL bottles) and were required to drink it all, and they were prohibited from drinking anything other than the water provided.

All participants used the same equipment (golf clubs and balls) during the two rounds. During the round, the participants carried their golf equipment in a cart and walked. The scores were tallied during the round. A putting performance test was conducted at the end of the play on each hole. Driving performances were conducted before the start, after the 9th holes, and after the 18th holes. Cognitive performance was conducted every three holes (before start and after 3th, 6th, 9th, 12th, 15th, and 18th holes).

### 2.4. Measures and Data Collection

#### 2.4.1. Interstitial Glucose

The glucose concentration in the interstitial fluid closely correlates with the glucose concentration in the blood [13,14]. Thus, in this study, interstitial glucose was used to evaluate blood glucose levels. Interstitial glucose levels were measured by the CGM system (Freestyle Libre; Abbott Diabetes Care, Alameda, CA, USA). This system continuously measures the glucose concentration in the interstitial fluid collected from cells immediately below the skin, and its details have been reported elsewhere [16,20].

#### 2.4.2. Golf Performance

Golf performance was evaluated by strokes, putting performance tests, and driving performance tests.

1. Scores

The scores were assessed to determine the difference between the participant’s number of strokes and the predetermined number of strokes for each hole. Strokes were recorded by participant self-report and accompanying research staff. Participants reported their strokes after checking with the research staff for errors.

2. Putting performance

Putting performance was evaluated on the percentage of successful putts that each participant made from the designated distance (2.5 m). Testing was performed at the end of each hole in which it was completed. The research staff determined the location of the putt. To eliminate the influence of technology, the location had to be as flat and straight as possible. The putters and balls were those used by each participant in the play. No practice was performed, and one measurement was performed. Success was achieved if a player made the cup in one stroke.

3. Driving performance

Driving performance was evaluated on three items: club head speed (CHS), distance, and accuracy. Driving performances were made using TrackMan 4 (TrackMan Japan Inc., Tokyo, Japan). The TrackMan 4 is used by low- and high-handicap golfers based on its accuracy [21,22]. The monitor was prepared according to the manufacturer’s requirements, which consisted of positioning it 2.8 m behind the impact area. In the test, the participants used their own driver and the same practice ball (DISTANCE 350, Sumitomo Rubber Industries, Ltd., Hyogo, Japan). Participants were permitted to complete a self-defined number of practice swings and hit balls until they felt ready to begin testing.

Tests were conducted in the starting order. Five driver shots were performed, and participants rested between shots for 60 s; the testing session of each participant lasted for 10 min. Participants were blinded to their values from the monitor.

#### 2.4.3. Cognitive Performance

Cognitive performance was evaluated using an adapted visual analog scale (VAS) questionnaire [23], which has been used in previous studies. The following three items were evaluated: self-perceived levels of fatigue (PLF), self-perceived levels of concentration (PLC), and self-perceived levels of relaxation (PLR).

Participants completed a VAS questionnaire (a questionnaire consisting of words describing minimum/maximum status marked at the left and right ends of a 100 mm long line drawn horizontally).

#### 2.4.4. Other Variables

Age and status of participation in the competition were self-reported. Body height was measured using a stadiometer (YHS-200D, YAGAMI Inc., Nagoya, Japan). Bodyweight and body fat percentage were measured by bioelectrical impedance analysis (InBody 470, InBody Japan Inc., Tokyo, Japan). Body height (to the nearest 0.1 cm), bodyweight (to the nearest 0.1 kg), and body fat percentage (to the nearest 0.1%) were measured with the participants wearing lightweight outdoor clothes without shoes.

Weather conditions were recorded for each trial date, including ambient temperature, wind speed, wind direction, and precipitation. Data were collected from the Hatoyama Meteorological Agency Observatory, which is located near the golf course [24]. Putting green conditions that were recorded for each trial included cutting heights, green speed, and compaction. Data were collected by the Ishizaka Golf Club.

#### 2.4.5. Data Analysis

The CGM systems have an inevitable time delay, which is relevant under exercise [25]. Thus, in this study, the average value was calculated for every three holes from the interstitial glucose level measured in each hole, and that value was used for the analysis. The score and putting success rate were calculated for each of the three holes and used as the analysis value. The driving performance, which was the median of the five shots, was used as the analysis value. Cognitive performance was defined as the distance (mm) from the left side of the line to the mark and was used for the analysis.

#### 2.4.6. Statistical Analyses

All statistical analyses were performed using JMP version 14.3.0 (SAS Institute Inc., Cary, NC, USA). All data are expressed as mean ± standard deviation. Data normality was evaluated using the Shapiro–Wilk test. A two-way analysis of variance (trial × time) with repetition was used to compare measurements across trials. Eta squared (η^2^) was determined as a measure of effect size for the two-way analysis. If the main effect of enforcement was found, post hoc analyses were conducted with the paired *t*-test. Cohen’s d was determined as a measure of effect size for *t*-test comparisons. All reported *p*-values were two-tailed, and *p*-values < 0.05 were considered statistically significant.

## 3. Results

### 3.1. Weather and Putting Green Conditions

Each trial date was similar, no rainfall was recorded, the ambient temperature was around the average temperature for March (7.5 °C), the average wind speed was around the average speed for March (1.9 mph), and the wind direction was north–northwest for both days [25] (Table 1). Green speed and compaction were typical for each test date. The putting green conditions did not differ significantly for both days.

### 3.2. Interstitial Glucose

**Figure 2:**
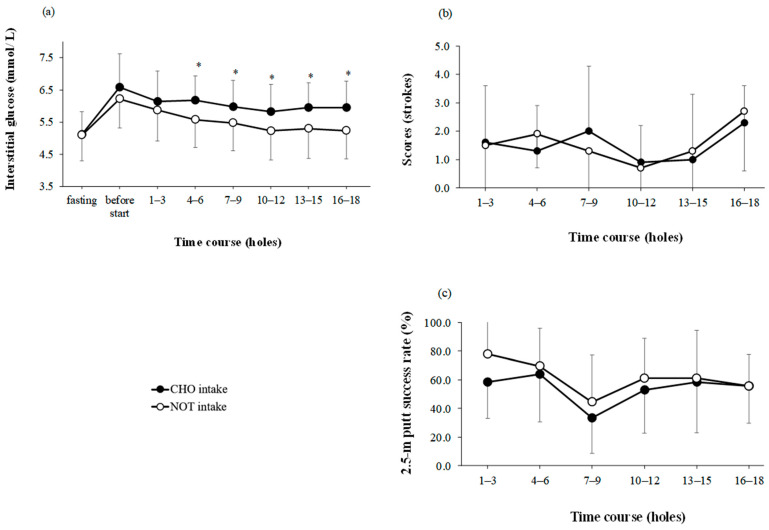
(**a**) Interstitial glucose changes, (**b**) scores and (**c**) 2.5 m putt success rate during a round of golf with carbohydrate-supplemented gummies continuously ingested (CHO intake) or noted (NOT intake). Data are presented as means ± SD. * significant difference between groups at the same point (*p* < 0.05).

There were main effects for trial and time for interstitial glucose (trial: *p* < 0.001, time: *p* < 0.001; interaction: *p* = 0.923; η^2^ = 1.604; Figure 2a). The interstitial glucose level was significantly higher in the CHO intake than in the NOT intake at the 4th–6th; *p* = 0.042; d = 1.412, 7th–9th; *p* = 0.046; d = 1.292, 10th–12th; *p* = 0.037; d = 1.481, 13th–15th; *p* = 0.020; d = 1.695, and 16th–18th holes; *p* = 0.023; d = 1.518.

### 3.3. Golf Performance

The scores were significant for the main effects of the trial; however, significance for the main effects of time was noted (trial: *p* = 0.906, time: *p* = 0.148, interaction: *p* = 0.882; η^2^ = −0.032; Figure 2b). The 2.5-m putt success rate was not significant for the main effects of the trial; however, significance for the main effects of time was noted (trial: *p* = 0.101, time: *p* = 0.009, interaction: *p* = 0.892; η^2^ = −0.200; Figure 2c).

**Figure 3:**
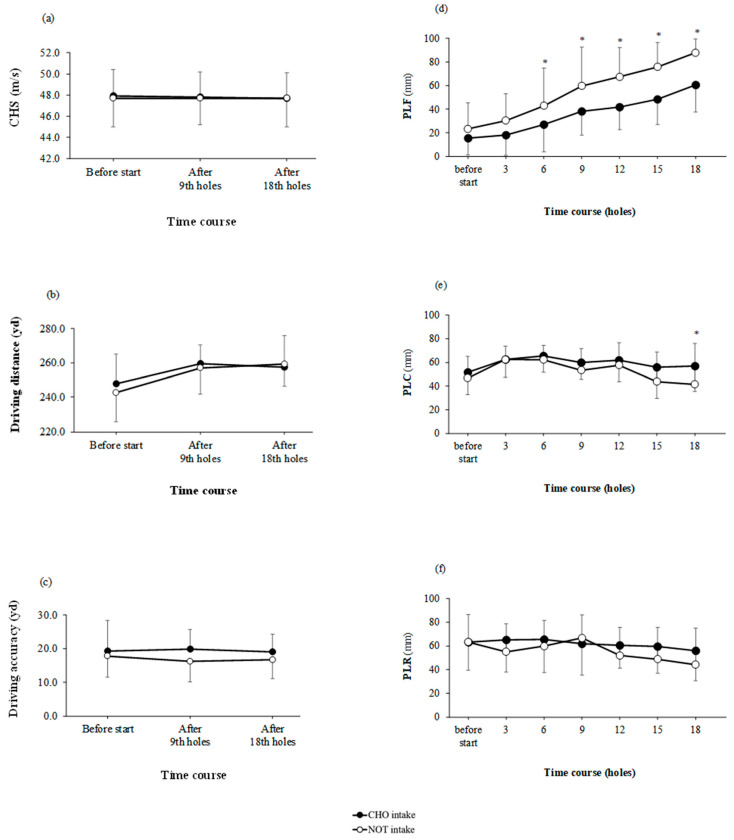
(**a**) Club head speed (CHS), (**b**) driving distance, (**c**) driving accuracy, (**d**) self-perceived levels of fatigue (PLF), (**e**) self-perceived levels of concentration (PLC), and (**f**) self-perceived levels of relaxation (PLR) during a round of golf with carbohydrate-supplemented gummies continuously ingested (CHO intake) or not (NOT intake). Data are presented as means ± SD. * significant difference between groups at the same point (*p* < 0.05).

The driving distance was not significant for the main effects of the trial; however, significance for the main effects of time was found (trial: *p* = 0.575, time: *p* = 0.005, interaction: *p* = 0.743; η^2^ = 0.263; Figure 3b). CHS was not significant for the main effects of trial and time interactions (trial: *p* = 0.818, time: *p* = 0.987, interaction: *p* = 0.992; η^2^ = −0.033; Figure 3a). The driving accuracy measurements were not significant for the main effects of trial and time interactions. (trial: *p* = 0.103, time: *p* = 0.928: interaction: *p* = 0.834; η^2^ = 0.900; Figure 3c).

### 3.4. Cognitive Performance

PLF was a main effect of the trial and time (trial: *p* < 0.001, time: *p* < 0.001, interaction: *p* = 0.657; η^2^ = 1.990; Figure 3d). PLF was significantly lower in the CHO intake than in the NOT intake on the 6th; *p* = 0.047; d = 1.125; 9th; *p* = 0.043; d = 1.328, 12th; *p* = 0.001; d = 1.853, 15th; *p* = 0.022; d = 1.631, and 18th holes; *p* = 0.017; d = 1.694.

There were main effects for trial and time for PLC (trial: *p* = 0.032, time: *p* = 0.024, interaction: *p* = 0.835; η^2^ = 1.314; Figure 3e). PLC was significantly higher in the CHO intake than in the NOT intake on all 18 holes; *p* = 0.044; d = 1.211.

The value of the CHO intake during the golf round was higher than that of the start. PLR was not significant for the main effects of trial and time interactions (trial: *p* = 0.121, time: *p* = 0.350, interaction: *p* = 0.889; η^2^ = 0.739; Figure 3f).

## 4. Discussion

In this study, we found that the continued carbohydrate intake with gummies during a round of golf, although not found to be effective on golf performance, may prevent the reduction of interstitial glucose levels and attenuated fatigue in the controlled study. Moreover, gummy intake had the potential to sustain concentration during a round of golf.

Continuous carbohydrate intake with gummies was found to prevent interstitial glucose decline on the 4th–6th, 7th–9th, 10th–12th, 13th–15th, and 16th holes. In addition, no decrease in interstitial glucose levels was observed in the second half of the round (Figure 2a). In a previous study, participants who ate each of the test meals showed a change in blood glucose levels in the second half of the golf round (9 holes), i.e., Stevenson et al. [4] reported −5.2% and Robinson reported −10.2% and −10.1%. On the contrary, in the present study, the rate of change in the interstitial glucose levels of the CHO intake in the second half of the golf round (9 holes) was +3.4%. This occurred because the test meal contained a sufficient amount of carbohydrates to maintain interstitial glucose levels in the second half of the golf round. The carbohydrate masses used in the previous studies were 48.8 g for Stevenson et al. (2009) [4] and 98 g for Robinson et al. (2018) [26]. By contrast, the test food used in the present study contained 162.5 g of carbohydrates (155.3 g of sugar), a much higher amount than those in previous studies. Moreover, consumption of carbohydrates at breakfast was considered to have influenced the results. Stevenson et al. (2009) [4] reported that participants who fasted and ate the zero-energy test meal experienced a change in blood glucose levels of −0.4 (−6.8%). On the contrary, in the present study, the rate of change in interstitial glucose levels in the NOT intake in the second half of the golf round (9 holes) was ± 0.0%.

In the present study, the participants ate a breakfast containing 1.02 kg BW of carbohydrates per kg BW. A previous study reported that if a pre-exercise carbohydrate-rich meal is combined with the ingestion of carbohydrates during exercise, then the improvements in endurance capacity during cycling [27] and running [28] tasks are greater than when either of these carbohydrate meals was adopted separately. The results of the present study support these findings and extended them.

Continuous carbohydrate intake with gummies was found to reduce the increase in PLF on the 6th, 9th, 12th, 15th, and 18th holes (Figure 3d). More interestingly, this study showed that simultaneous increase in fatigue level and decrease in interstitial glucose levels. Several previous studies have examined carbohydrates and supplements and levels of alertness, fatigue, and stress during golf rounds [4,24,29]. However, comparing previous studies is challenging as no previous studies have assessed the effects of carbohydrate intake on the PLF, PLC, and PLR during golf under standardized conditions. Previous studies have also shown that hypoglycemia (<3.6 mmol/L) is associated with higher ratings of self-perceived fatigue during a golf round [12]. Although hypoglycemia was not observed in this study, a decrease in blood glucose level and an increase in fatigue level were observed simultaneously. Previous studies have not examined the association between lower interstitial glucose levels and increased fatigue levels. At this time, identifying the mechanism is challenging; thus, further research utilizing the CGM system as used in this study is warranted.

Moreover, continuous carbohydrate intake with gummies was found to reduce the decrease in PLC on all 18 holes (Figure 3e). Furthermore, this study showed that a decrease in the degree of concentration was seen sometime after a decrease in interstitial glucose levels and an increase in fatigue. Thus, our results show that continuous carbohydrate intake during the first half of the golf round attenuated the fatigue level after the middle of the golf round and maintained the concentration level after the second golf round.

In this study, the continuous intake of carbohydrates with gummies did not affect driving performance. Previous studies have reported an association between fatigue and decreased driving performance using various test diets [24,29,30]; in the present study, we hypothesized that fatigue attenuation would prevent a decline in driving performance. However, the results of the present study contradict those of previous studies. It is possible that the study did not adequately elicit fatigue in the participants. Our study participants usually played many rounds of golf because one round was not fatiguing enough to affect performance. In addition, this study did not adequately bring out the competitiveness of the participants. Previous studies have offered prizes based on rankings to increase competitiveness and elicit fatigue among participants [24]. Therefore, a research protocol that can fully elicit fatigue in participants is necessary.

In this study, the 2.5 m putt success rate was not significant for the main effects of trial and time (Figure 2c). Previous studies did not provide a unified view of the effect of fatigue on putting performance [4,5]. The results of this study support the findings of Hayes et al. (2009) [5] that fatigue does not affect putting performance. However, we cannot conclude from the results of this study that fatigue is not related to putting performance. Therefore, conducting standardized putting performance tests and accumulating evidence in the future are imperative.

In this study, scores were not significant for the main effects of trial and time. On the USPGA Tour, 61–87% of the scores can be explained by driving distance, fairway percentage, par on percentage, and putts made [31,32]. In this study, it was not possible to measure fairway percentage or par on percentage. Therefore, it cannot be said that the continuous intake of carbohydrates does not affect the scores in this study. In the future, it will be necessary to collect data on ball hodgepodge related to scores to verify the relevance. The total distance of the blue tees used in this study was 6550 yards, which is less for male university student golfers. Furthermore, the tests were conducted in February and March, and the rough was not long. Also, these are also factors that mean the missed shots may not have affected the score.

A main effect of time was noted on putting success (Figure 2c). This may be related to the fact that the success rate for the 17 holes was lower than that for others. The putting success rate for all holes was 46.8%; however, the success rate for eight holes was as low as 12.5%. Therefore, the main effect of time was found in the putting success rate.

A main effect of time was found on the driving distance (Figure 3b). The driving distance was reported to be significantly associated with CHS and smash factor [33]. The smash factor is ((initial ball speed)/(CHS at impact)). Participants had a learning effect in the smash factor: therefore, in this study, the driving distance was affected by the increase in the smash factor.

To the best of our knowledge, this is the first study whose results demonstrate that continuous intake of carbohydrates during a round of golf attenuated the decline in interstitial glucose level and prevented fatigue from the middle of the golf round, and concentrations were maintained in the last half of the round of golf. Furthermore, the results showed a simultaneous increase in the fatigue level and decrease in interstitial glucose levels. Therefore, as a practical application, competitive golfers should plan nutritional supplementation to maintain interstitial glucose during a round of golf. Although gummies were selected for this study because of their easy availability, this could be achieved using other foods, such as bread, rice balls, and dried fruits, which have high carbohydrate contents and are easy to consume. Competitive golfers should consume these carbohydrate-rich foods on a sustained basis to prevent fatigue when playing golf.

The strength of this study was attributed to the use of a standardized real-life application study design and on-course testing and measurement of interstitial glucose levels during a round of golf and anthropometric data of competitive golfers. This study had several limitations. First, this study was conducted with an open-study design, and no placebo was set. Placebo gummies with noncaloric artificial sweeteners were not ethically used in this study because they exceeded the maximum no-action dose. Therefore, the study design may have influenced the cognitive performance results to a small degree. Second, carbohydrate-supplemented gummies were used; however, the effect of chewing could not be eliminated. Previous studies have shown that chewing is associated with attenuated brain activity [34] and cognitive performance [35]. Third, the CGM system used has a slower rise time than blood sampling and generally lower peak glucose values, which may underestimate the effect of carbohydrate intake on the glucose response [36]. Fourth, small sample sizes are problematic regardless of the study outcome with issues ranging from inconclusive results and low precision to unrepeatable “discoveries” [37]. The results of this study should be interpreted with caution in light of these considerations.

## 5. Conclusions

In this study, continuous carbohydrate intake had no effects on golf performance. On the contrary, the continuous intake of carbohydrates during the golf round attenuated the decline in interstitial glucose level and prevented fatigue in the controlled trial from the middle of the golf round, and concentration was maintained in the last half of a round of golf. These findings suggest that continuous carbohydrate intake with gummies may be effective in attenuating fatigue and maintaining the concentration of competitive golfers. Thus, this study can propose nutritional strategies for competitive golf.

## Figures and Tables

**Table 1:** Weather and putting greens conditions on trial day.

	1st Day	2nd Day
Weather data		
Conditions	Sunny	Light Cloud
Mean ambient temperature (°C)	6.7	6.7
Mean wind speed (mph)	1.1	1.7
Wind direction	North–northwest	North–northwest
Precipitation (cm)	0	0
Putting greens date		
Cutting Heights (mm)	3.8	3.8
Greens speed (ft)	9.5	9.8
Compaction (mm)	6.7	6.7

## Data Availability

The underlying research materials related to this paper are available from the corresponding author upon request.
